# Bioabsorbable Bypass Grafts Biofunctionalised with RGD Have Enhanced Biophysical Properties and Endothelialisation Tested *In vivo*

**DOI:** 10.3389/fphar.2016.00136

**Published:** 2016-05-25

**Authors:** Larisa V. Antonova, Alexander M. Seifalian, Anton G. Kutikhin, Victoria V. Sevostyanova, Evgeniya O. Krivkina, Andrey V. Mironov, Andrey Y. Burago, Elena A. Velikanova, Vera G. Matveeva, Tatiana V. Glushkova, Evgeniya A. Sergeeva, Georgiy Y. Vasyukov, Yuliya A. Kudryavtseva, Olga L. Barbarash, Leonid S. Barbarash

**Affiliations:** ^1^Research Institute for Complex Issues of Cardiovascular DiseasesKemerovo, Russia; ^2^Centre for Nanotechnology and Regenerative Medicine, UCL Division of Surgery and Interventional Science, University College LondonLondon, UK; ^3^NanoRegMed LtdLondon, UK

**Keywords:** poly(3-hydroxybutyrate-co-3-hydroxyvalerate), poly(𝜀-caprolactone), vascular grafts, RGD peptides, morphology, physico-mechanical properties, endothelialisation, biocompatibility

## Abstract

Small diameter arterial bypass grafts are considered as unmet clinical need since the current grafts have poor patency of 25% within 5 years. We have developed a 3D scaffold manufactured from natural and synthetic biodegradable polymers, poly(3-hydroxybutyrate-co-3-hydroxyvalerate) (PHBV) and poly(𝜀-caprolactone) (PCL), respectively. Further to improve the biophysical properties as well as endothelialisation, the grafts were covalently conjugated with arginine-glycine-aspartic acid (RGD) bioactive peptides. The biophysical properties as well as endothelialisation of PHBV/PCL and PCL 2 mm diameter bypass grafts were assessed with and without biofunctionalisation with RGD peptides *in vitro* and *in vivo*. Morphology of the grafts was assessed by scanning electron microscopy, whereas physico-mechanical properties were evaluated using a physiological circulating system equipped with a state of art ultrasound vascular wall tracking system. Endothelialisation of the grafts *in vitro* and *in vivo* were assessed using a cell viability assay and rat abdominal aorta replacement model, respectively. The biofunctionalisation with RGD bioactive peptides decreased mean fiber diameter and mean pore area in PHBV/PCL grafts; however, this was not the case for PCL grafts. Both PHBV/PCL and PCL grafts with RGD peptides had lower durability compared to those without; these durability values were similar to those of internal mammary artery. Modification of PHBV/PCL and PCL grafts with RGD peptides increased endothelial cell viability *in vitro* by a factor of eight and enhanced the formation of an endothelial cell monolayer *in vivo* 1 month postimplantation. In conclusion, PHBV/PCL small-caliber graft can be a suitable 3D scaffold for the development of a tissue engineering arterial bypass graft.

## Introduction

Reconstructive surgery is a conventional treatment of coronary artery disease and peripheral artery disease, and autologous saphenous vein, internal mammary and radial artery grafts are commonly used (Taggart, 2013). The problem is that a significant proportion of the patients does not have suitable veins or arteries (Desai et al., 2011). Synthetic grafts prepared from poly(ethylene terephthalate) (PET, Dacron) or expanded poly(tetrafluoroethylene) (ePTFE, Teflon) were suggested as an appropriate option (Roll et al., 2008). Nevertheless, these grafts perform well as large-caliber replacements but their long-term patency is discouraging in small-caliber applications (<6 mm diameter) such as coronary, crural, or microvessel surgery (Chlupác et al., 2009). A reason for this is a low blood flow resulting in an intimal hyperplasia and/or thrombus formation at the anastomotic site (Chlupác et al., 2009).

Therefore, tissue engineering of vascular grafts is a promising approach for the replacement of small-caliber blood vessels (G et al., 2015). Small-caliber vascular grafts prepared from biodegradable polymers act as a scaffold for the formation of a new blood vessel *in situ* (G et al., 2015). It was previously demonstrated that poly(𝜀-caprolactone) (PCL), a synthetic biodegradable polymer, can be used for the fabrication of the vascular grafts (de Valence et al., 2012). Poly(3-hydroxybutyrate-co-3-hydroxyvalerate) (PHBV) is a natural biodegradable polymer synthesized by bacteria as a storage compound under growth limiting conditions (Quillaguamán et al., 2010). One of its monomers, 3-hydroxybutanoic acid, is a natural metabolite produced in the human body (Quillaguamán et al., 2010). This ensures a high biocompatibility of PHBV (Quillaguamán et al., 2010). Earlier studies by our group revealed that the combination of PHBV with PCL improves biocompatibility of electrospun small-caliber vascular grafts (Antonova et al., 2015a,b).

Arginine-glycine-aspartic acid (RGD) is a cell adhesion motif displayed on many extracellular matrix proteins such as fibronectin, laminin, vitronectin, fibrinogen, von Willebrand factor, and osteopontin (Wang et al., 2013). This motif is a crucial ligand for integrins, receptors responsible for cell attachment, migration, proliferation, differentiation, and survival (Harburger and Calderwood, 2009). Therefore, RGD-containing peptides, or simply RGD peptides, were suggested as a promising agent for the modification of polymers to improve their biocompatibility, particularly adhesive properties (Ren et al., 2015). We carried out this study with the aim to assess whether modification with RGD peptides affects morphology, physico-mechanical properties, and biocompatibility of PHBV/PCL and PCL vascular grafts.

## Materials and Methods

### Graft Preparation

Small-caliber vascular grafts were fabricated by electrospinning (Nanon-01A, MECC) from PCL (80,000 kDa, 14%)/chloroform or PHBV/PCL (5:10%)/chloroform solutions using the following respective parameters: 15 or 20 kV voltage, 0.5 or 0.3 mL/h feed rate, 2 mm rotating drum diameter, 22G needle, and 150 mm tip-to-collector distance. All reagents unless otherwise stated were purchased from Sigma–Aldrich.

### Polymer Amination-Activation

A 1:1 2-propanol: water solution was used to remove oil and dirt residues from the polymers, which were then washed thoroughly with deionised water (Causa et al., 2010). Grafts were treated with 10% ethylenediamine (EDA) – 2-propanol solution at 37°C for 1 h (Zhang and Hollister, 2009). After treatment, the samples were rinsed with 0.3% Tween-20 solution in deionised water until all amination solution was washed away or until the samples sank in water (Causa et al., 2010). Samples were then air dried.

### RGD Peptide Conjugation

For the linker solution, 0.6 mg/mL 1-ethyl-3-(3-dimethyla minopropyl) carbodiimide, 0.827 mg/mL *N*-hydroxysuccini mide, and 3.59 mg/mL succinic acid were combined in phosphate buffered saline (PBS) and roll-mixed for 1 h (Gabriel et al., 2006). The synthesis of the lauric acid, glycine, arginine, glycine, aspartic acid, glycine, aminohexanoic acid (LA-GRGDG-AHex) – RGD containing biomolecule was previously described in detail (Sedaghati et al., 2014). The peptide was diluted to the appropriate concentration (0.2 mg/mL) in PBS with 0.05% Tween-20 (PBST) (Zhang and Hollister, 2009).

Samples were treated with the linker solution for 30 min in the dark, and after a thorough wash with PBS, they were incubated with the RGD solution on a shaker for 1 h in the dark (37°C), and then were put in the fridge overnight (4°C). After allowing for the reaction to proceed, samples were washed thoroughly with PBST, PBS, and then air dried (Gabriel et al., 2006).

### Amino Acid Detection

To identify whether conjugation with RGD peptides was successful, we performed Orange II staining and ninhydrin test. For Orange II staining, the polymer samples were immersed in 1.5 mL of Orange II sodium dye solution (Noel et al., 2011). Briefly, 14 mg/mL of Orange II dye were dissolved in a pH 3 solution of hydrochloric acid (HCl). Incubation for 30 min at 37°C followed after the samples were immersed in dye solution. The samples were rinsed well in HCl solution (pH 3) to remove unbound dye. After drying in air, the samples were immersed in a 1 mL of sodium hydroxide (NaOH, pH 12) alkaline solution to free the dye in solution. Absorbance at 480 nm of the dye containing solution was measured using the multiwell plate reader in the UCL Division of Surgery and Interventional Science (Royal Free Hospital, London). For ninhydrin test, each sample was immersed in a 0.178 mg/mL solution of ninhydrin in ethanol for 1 min, incubated at 37°C for 30 min in order to react, and then 2 mL/sample of tetrahydrofuran was used to dissolve the samples. To each dissolved sample, 2 mL of 2-propanol was added and the absorbance of the final solution was measured at 560 nm (Santiago et al., 2006).

To determine the effect of RGD peptides on polymer surface, we used Fourier transform infrared spectroscopy (FTIR). Following the colorimetric test, a more specific analysis of the FTIR provides information specific for the RGD peptides which is further evidence of their covalent immobilization on the polymer. Measurements were taken twice to confirm that the spectra were identical for unmodified and aminolysed PHBV/PCL and PCL.

The individual amino acids of the synthesized RGD peptides were identified by thin layer chromatography (TLC). Sample tubes were placed in 20.2% w/w HCl acid (concentrated) solution in sealed autoclave-able containers with enough HCl so as to just float off the surface. Bottles were placed in the oven (105°C) overnight. The sample containing HCl solution was evaporated off and 100 μL distilled water was added after the containers cooled down. TLC was performed using F254 silica-gel-on-glass plates (Merck). A butanol:acetic acid:water 1:1:1 mixture was used as developing solvent and after 30 min the dried plates were sprayed with a 0.2% ninhydrin solution to detect the spots. The process was repeated twice and Rf factors were calculated from the center of each spot according to the equation:

Rf = Da/Ds,

where Da is the distance traveled by the analyte and Ds is the distance traveled by the solvent. An arginine/aspartic acid (1 mg/mL in deionised water) solution was also impregnated into the plate to compare with the dissolved sample. Reference for Rf values were taken from the previous study (Sedaghati et al., 2014).

### Morphological Assessment

PHBV/PCL and PCL graft samples 0.5 mm × 0.5 mm with and without RGD peptides (*n* = 5 per each group) were examined using scanning electron microscopy (Hitachi S-3400N, Hitachi) with Au-Pd sputter coating (Quorum Technologies) of 30 nm thickness. Fiber diameter, pore area, and porosity were measured using ImageJ (National Institutes of Health). Mean fiber diameter and mean pore area were calculated after at least 100 measurements per each sample.

### Evaluation of Physico-Mechanical Properties

Assessment of durability and elastic deformation properties was performed using universal testing machine (Zwick/Roell). Testing was performed with 1 cm working segment length, 0.01 N preload, and 10 mm/min crosshead speed. Durability, elasticity, and stiffness were evaluated by yield stress, relative elongation, and elastic modulus, respectively. We assessed 6 vascular grafts per each group. An ePTFE graft, human saphenous vein (SV) and internal mammary artery (IMA) (*n* = 6 per each) were used as the controls. SV and IMA were collected from patients who underwent coronary artery bypass graft surgery, and all the participants provided written informed consent after receiving a full explanation of the study. The study protocol was approved by a local ethical committee.

**FIGURE 1:**
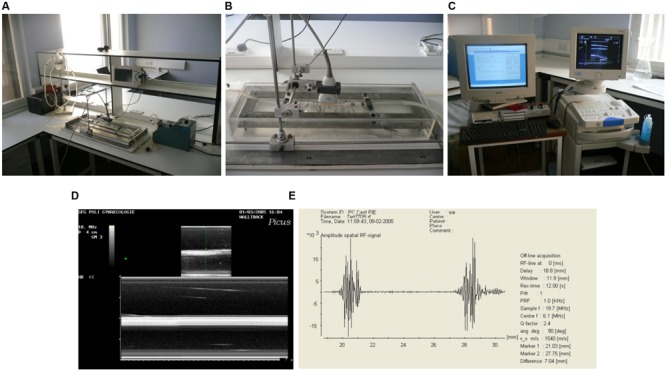
**Arterial pulsatile flow circuit. (A)** Photograph of the flow circuit apparatus. **(B)** Detail of the perspex/saline bath and ultrasound probe in flow circuit. **(C)** Photograph of the ultrasound machine and wall tracking system. **(D)** B-mode (top) and M-mode (bottom) ultrasound images of the graft mounted in flow circuit. **(E)** Signal generated by anterior and posterior walls of the graft.

With the aim to assess compliance, defined as the change in volume of a structure with respect to the change in pressure, a flow circuit was set up as shown in **Figure 1** in order to perfuse grafts. This circuit was designed to create arterial pulsatile flow of physiological pressures and characteristics. A pulsatile blood pump (Harvard Apparatus) was used to create pulsatile flow, with a pulse rate of 60 beats per minute. This was connected by a flexible plastic tubing via the grafts and vessels under testing to a fluid reservoir (Radnoti Glass Technology), which in turn was connected back to the pump, thus creating a circuit. A heat exchanger (Grant Instruments) maintained the fluid reservoir at 37°C. The pressure of the circuit was measured using a pressure transducer (TranStar Pressure Monitoring System) and pressure monitor (Datex Engstrom, Datex-Ohmeda Division Instrumentarium), connected to the circuit distal to the grafts. Pressure was varied by altering the level of the fluid reservoir above the grafts. Heparinised (Heparin sodium, CP Pharmaceuticals Ltd., Wrexham, UK) whole human blood mixed with normal saline (NaCl 0.9%, Baxter Healthcare) in a ratio of 2:1 respectively, was used to perfuse the circuit. For each pint of blood used, approximately 2 mL (1000 I.U. in 1 mL) of heparin was added.

Fifty millimeter lengths of two different grafts were used (*n* = 6). Grafts were connected one at a time into the flow circuit and positioned in a bath of normal saline. All conduits were subjected to 3% longitudinal stretch to reproduce *in vivo* characteristics (Sarkar et al., 2007). In order to measure wall distension due to the pressure in the circuit, a duplex color flow ultrasound system (Picus, Pie Medical Systems) was used to obtain real time M-mode images of each segment. A specially designed supporting frame was used to hold the 10 MHz linear array probe in the bath directly over the segment, so reliable readings could be taken. At each mean pressure wall distension was found from end-diastolic and end-systolic intraluminal diameters for each segment, determined by a wall-tracking system (Pie Medical Wall Track Version 2, Pie Medical Systems). Mean pressures of 30–90 mmHg were used, increasing at 30 mmHg increments. At each pressure, wall movement was measured at three sites on each graft for a period of 12 s.

After the collection of data, compliance (C) was calculated from wall distension and pressure, using the appropriate equation (Hermeling et al., 2012; Ahmed et al., 2014):

$$\text{C}(\%{\text{mmHg}}^{-}{}^{1}\times 10^{-}{}^{2})=((\text{Ds-Dd})\text{/Dd}\times {\text{10}}^{\text{4}})\text{/}(\text{Ps-Pd}),$$

where P, D, s, and d are pressure, diameter, systole, and diastole respectively.

At each of the three locations, six measurements of wall distension were made over the 12 s, and average data were calculated.

### Cell Viability Assay

Human umbilical vein endothelial cells (HUVECs) were isolated according to adapted protocol of Jaffe et al. (1973). Cells were cultured using endothelial cell culture medium kit (BD Bioscience). For the experiments, we used cells of 5th passage. Briefly, 1.8 mm diameter PHBV/PCL, PCL, PHBV/PCL/RGD, and PCL/RGD scaffolds (*n* = 6 per each group) were fixed at the bottom of the wells of 24-well plate using 0.6% agarose (Amresco). Cells (2.5*10^5^ cells per polymer sample) were cultured during 6 days at 37°C, 5% CO_2_, and high humidity (MCO-18AIC, Sanyo). After 6 days of culture, we stained cells with Hoechst 33342 (2 μg/mL) and PKH26 (2 μg/mL) or ethidium bromide (3 μg/mL) and acridine orange (1 μg/mL), and then assessed cell viability by fluorescence and confocal laser scanning microscopy (AxioObserver.Z1 and LSM 700, respectively, Carl Zeiss). Cells were counted in 10 visual fields (x200) per each sample using ImageJ with the further calculation of total and dead cell count per 1 mm^2^. This experiment was performed in triplicate.

### *In vivo* Implantation

All animal experiments were performed in 6-month-old male Wistar rats (400–450 g body weight, 12–14 weeks of age, *n* = 20) according to all official and ethical requirements. The animals were allocated in polypropylene cages (five animals per cage) lined with wood chips and had access to water and food (rat chow) *ad libitum.* Throughout the whole time of experiment, standard conditions of temperature (24 ± 1°C), relative humidity (55 ± 10%), and 12 h light/dark cycles were carefully maintained, and the health status of all rats was monitored daily.

After ethylene oxide sterilization, 2 mm inner diameter and 10 mm length PHBV/PCL and PCL grafts with and without RGD peptides grafts were implanted into rat abdominal aorta (*n* = 5 per each group) after the induction of anesthesia with 3% isoflurane. During the surgery, all animals received inhalation anesthesia with 1.5% isoflurane. Briefly, a midline laparotomy was performed. After the isolation of the abdominal cavity with a sterile cloth, intestinal loops were moved to the right and wrapped in a wet warm cloth. The posterior peritoneal leaflet was opened along the mesenteric root, and aorta was mobilized from the level of renal arteries to bifurcation. Then, aorta was temporarily occluded with two microvascular bulldog clamps distally from the renal arteries and proximally from the bifurcation. Aorta and vena cava inferior were occluded in parallel. Both proximal and distal anastomoses were performed using Prolene 8–0. Graft was washed between the performances of these anastomoses. After the implantation, the anterior abdominal wall was closed layer-by-layer with a blanket suture (4–0 or 2–0 Vicryl). All procedures were performed using strict aseptic technique. All the grafts were implanted for 1 month.

After the explantation, grafts with the adjacent aortic tissue were fixed in 10% (w/v) neutral phosphate buffered formalin (Electron Microscopy Sciences) during 24 h at 4°C for the further histological and immunohistochemical examination.

### Histological Examination

Formalin-fixed grafts were dehydrated in isopropanol during 30 h at 4°C, rinsed in distilled water, embedded in paraffin (Electron Microscopy Sciences), sectioned (5 μm), and finally mounted on glass microscope slides. For a deparaffinization, paraffin-embedded tissue sections were heated in dry oven at 60°C for 20 min, immersed in the following reagents: 3x xylene (Electron Microscopy Sciences) for 10 min, 100%, 95%, 70%, 50%, 30% ethanol for 1 min each, physiological saline for 2 min, PBS for 2 min, and finally rinsed with tap water.

For hematoxylin and eosin (H&E) staining, the sections were immersed in Harris Hematoxylin solution (Electron Microscopy Sciences) for 1 min, rinsed with tap water, immersed in 1% aqueous Eosin Y solution (Electron Microscopy Sciences) for 1 min, rinsed with tap water, dehydrated in ascending ethanol solutions (50%, 70%, 80%, 2x 95%, and 2x 100%), and then cleared 2x with xylene. For van Gieson staining, the sections were immersed in Weigert’s working hematoxylin solution (Electron Microscopy Sciences) for 10 min, rinsed with tap water, immersed in van Gieson stain (Electron Microscopy Sciences) for 3 min, rinsed with distilled water, dehydrated in ascending ethanol solutions (2x 95% and 2x 100%), and cleared 2x with xylene.

Coverslips were mounted onto a labeled glass slide with Permount (Electron Microscopy Sciences). After the staining, sections were evaluated by light microscopy (Axio Imager A1, Carl Zeiss) in a blinded fashion; three sections per stain were assessed from each rat (nine sections per rat).

### Immunohistochemistry

For immunohistochemical assessment, we used the kit of Leica Biosystems and rabbit anti-CD31 antibodies (Spring Bioscience) according to manufacturer’s instructions. Briefly, samples were boiled in citrate buffer (0.01 M, pH 6.0) for antigen retrieval, treated by the inhibitor of endogenous peroxidase for 10 min, and washed twice in phosphate buffer (0.01 M, pH 7.4) for 5 min. For blocking non-specific background staining, we treated the samples with Protein Block for 10 min with the further 2x washing in phosphate buffer for 5 min. Then, we stained the samples with 50 μL of primary anti-CD31 (PECAM-1) antibodies, incubated them in a wet chamber for 1 h, and performed the staining with secondary antibodies. After the washing in phosphate buffer, we treated samples with streptavidin-peroxidase conjugate and diaminobenzidine with the further assessment of the reaction which was stopped by cold distilled water. Slides were then stained with Mayer’s Hematoxylin and finally mounted. Native blood vessels and antibody diluent were used as a positive and negative control, respectively.

### Statistical Analysis

Statistical analysis was performed using GraphPad Prism (GraphPad Software). A sampling distribution was assessed by D’Agostino-Pearson test and Kolmogorov–Smirnov test. Depending on the distribution, descriptive data were represented by median and interquartile range (25th and 75th percentiles) or mean and standard deviation of the mean. Two independent groups were compared by Mann–Whitney *U*-test or two-tailed Student’s *t*-test. Independent groups numbering three or more were compared using Kruskal–Wallis test or analysis of variance (ANOVA), with pairs further compared by Mann–Whitney *U*-test or two-tailed Student’s *t*-test if statistically significant differences were revealed by Kruskal–Wallis test or ANOVA, respectively. An adjustment for multiple comparisons was performed using false discovery rate (FDR). *P*-values, or *q*-values if FDR was applied (*q*-values are the name given to the adjusted *p*-values found using an optimized FDR approach), ≤0.05 were regarded as statistically significant.

## Results

### PHBV/PCL and PCL Vascular Grafts Can Be Successfully Modified with RGD Peptides

**FIGURE 2:**
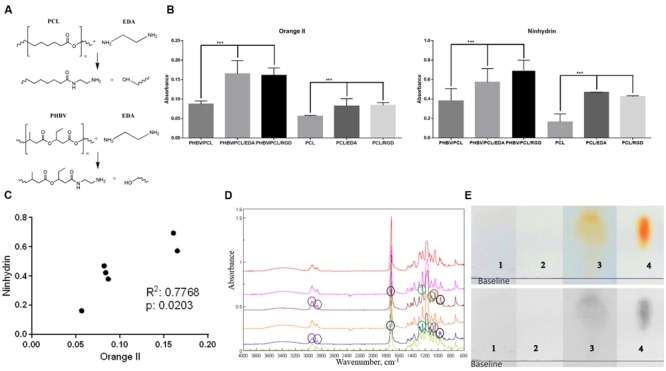
**Modification of PHBV/PCL and PCL vascular grafts with RGD peptides. (A)** Aminolysis reaction scheme between EDA, PHBV, and PCL monomers. **(B)** Orange II staining and ninhydrin test demonstrated increased amino group presence in PHBV/PCL and PCL grafts with EDA or RGD peptides compared to those without, data are represented as mean with standard deviation, ^∗∗∗^*P* < 0.001, two-tailed Student’s *t*-test. **(C)** Concordance test revealed a strong correlation between Orange II and ninhydrin assays for PHBV/PCL and PCL grafts with and without EDA treatment. **(D)** Fourier transform infrared spectroscopy showed the intensified signal from N–H stretching (around 960 cm^-1^, black circles) and the increase in amino residues in PHBV/PCL and PCL grafts with RGD peptides (brown and blue graphs, respectively). Peak around 1045 cm^-1^ (red circles) corresponds to Si-O-Si stretching, peaks around 1172 and 1240 cm^-1^ (green circles) correspond to C–O ester stretching, peak around 1720 cm^-1^ (blue circles) corresponds to C = O aliphatic ester stretching, and peaks around 2865 and 2941 cm^-1^ (violet circles) correspond to C–H asymmetric alkyl stretching. **(E)** Thin layer chromatography (TLC) spots after analyzing hydrolysed (1) PHBV/PCL, (2) PHBV/PCL/EDA, (3) PHBV/PCL/RGD, and (4) arginine and aspartic acid mixture on chromatographic silica to show the peptide content only in the RGD modified sample.

Primary amino groups were introduced to the inner surface of PHBV/PCL and PCL grafts via aminolysis with EDA (**Figure 2A**). Orange II treatment detected all primary amines on the unmodified PHBV/PCL and PCL grafts, after they were aminolysed, and after the linking reaction with the RGD peptides. There was an expected and statistically significant difference between the modified and unmodified polymer samples (**Figure 2B**). Ninhydrin testing confirmed the increase in amino group concentration from unmodified to RGD modified samples (**Figure 2B**), and the correlation of the results between the two tests was significant and strong (**Figure 2C**). Concentrations of amino groups were highest in PHBV/PCL/RGD (14.9 ± 2.9 μM/mm^2^) and PHBV/PCL/EDA (14.5 ± 0.5 μM/mm^2^) with equivalent concentrations on PCL samples reaching only half (7.55 ± 0.2 μM/mm^2^). The colorimetric tests showed the creation of amine groups on the polymers and provided a method of calculating a sample concentration of amino groups.

Fourier transform infrared spectroscopy showed that the 2° amine peak for RGD-modified PHBV/PCL was stronger than in non-modified equivalents. Added peptide species on the polymer were picked up and intensified the signal for the N–H wag which was visible at approximately 960 cm^-1^ (**Figure 2D**). The characteristic peak for lauric acid at approximately 1730 cm^-1^ was hidden by the strong ester carbonyl peak presented on PHBV as well as on PCL. The strong peak at approximately 1170 cm^-1^ was assigned to C–O–C stretching as ester residues were abundant in the polymer.

Finally, TLC demonstrated that sample separated into a spot with its highest and middle point Rf values very similar to those obtained from the arginine/aspartic acid sample, thus showing the content in those two amino acids and consequently the LA-GRGDC-AHex peptide we intended to attach to it (**Figure 2E**).

### RGD Peptides Alter Porous Structure of PHBV/PCL and PCL Vascular Grafts

**FIGURE 3:**
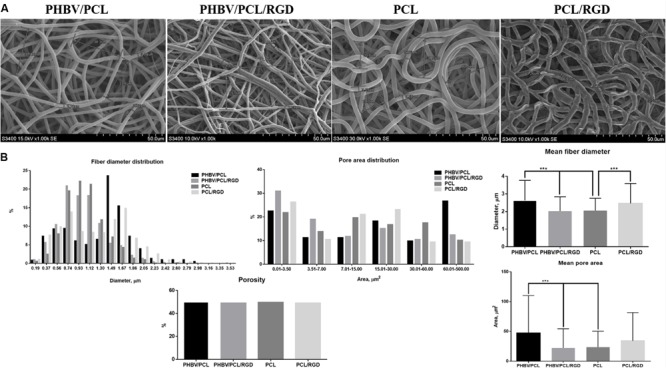
**PHBV/PCL and PCL vascular grafts with RGD peptides have a distinct morphology. (A)** Scanning electron microscopy images of PHBV/PCL and PCL grafts with and without RGD peptides. **(B)** Morphological parameters of PHBV/PCL and PCL grafts with and without RGD peptides; quantitative image analysis revealed a higher mean fiber diameter and mean pore area in PHBV/PCL compared to PCL grafts and in PHBV/PCL grafts with RGD peptides compared to those without, data are represented as mean with standard deviation, ^∗∗∗^*P* < 0.001, two-tailed Student’s *t*-test.

Both PHBV/PCL and PCL grafts had a highly porous structure with a respective 170–200 and 250–350 μm wall thickness and homogenous fibers (**Figure 3A**). Mean fiber diameter and mean pore area in PHBV/PCL grafts were significantly higher compared to PCL grafts (2.63 ± 1.14 and 2.06 ± 0.69 μm; 47.13 ± 63.00 and 23.59 ± 26.80 μm^2^, respectively). Modification with RGD peptides significantly decreased mean fiber diameter and mean pore area in PHBV/PCL grafts but increased mean fiber diameter in PCL grafts (**Figure 3B**). All the grafts had similar porosity around 50%.

### RGD Peptides Improve Physico-mechanical Properties of PHBV/PCL and PCL Vascular Grafts

**FIGURE 4:**
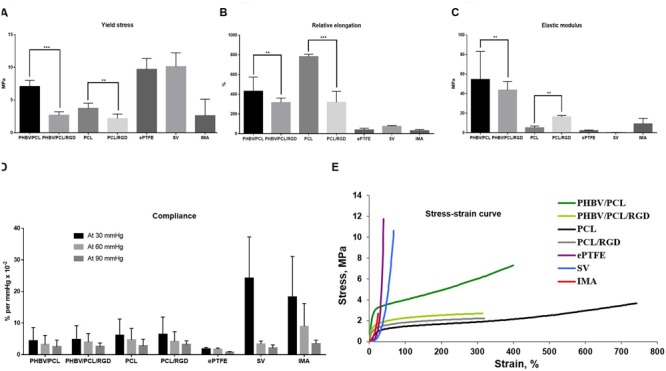
**PHBV/PCL and PCL vascular grafts with RGD peptides have improved physico-mechanical properties. (A)** Modification with RGD peptides decreased durability of PHBV/PCL and PCL grafts almost to the values of internal mammary artery (IMA). **(B)** Modification with RGD peptides reduced elasticity of PHBV/PCL and PCL grafts; however, it was still far from that of IMA. **(C)** Modification with RGD peptides decreased stiffness of PHBV/PCL grafts but increased stiffness of PCL grafts; for PCL grafts, stiffness was close to that of IMA. **(D)** Measurement of compliance found no significant differences for both PHBV/PCL and PCL grafts with and without RGD peptides. **(E)** Calculation of stress-strain curve of PHBV/PCL and PCL grafts with and without RGD peptides revealed it is still far from that of IMA. Data are represented as median with interquartile range, ^∗∗^*P* < 0.01, ^∗∗∗^*P* < 0.001, Mann–Whitney *U*-test.

Modification of PHBV/PCL and PCL scaffolds with RGD peptides significantly reduced durability of the scaffolds, making it similar to IMA (**Figure 4A**). It also decreased elasticity of both PHBV/PCL and PCL scaffolds (**Figure 4B**), and reduced stiffness of PHBV/PCL scaffolds but increased stiffness of PCL scaffolds (**Figure 4C**). However, RGD peptides had no effect on compliance (**Figure 4D**) and did not affect stress-strain curve (**Figure 4E**). Comparison of both polymer scaffolds with and without RGD peptides, ePTFE, SV, and IMA revealed that modification with RGD peptides made both types of polymer scaffolds more similar to IMA.

### RGD Peptides Increase Viability of Endothelial Cells Cultured on PHBV/PCL and PCL Scaffolds

**FIGURE 5:**
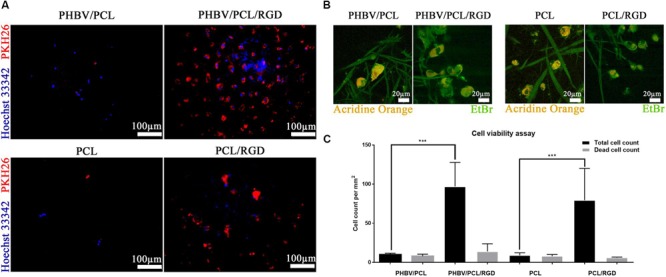
**PHBV/PCL and PCL scaffolds with RGD peptides improve cell viability. (A)** Fluorescence microscopy showed several-fold increase in total cell count on PHBV/PCL and PCL scaffolds with RGD peptides in comparison with those without. Nuclei and cytoplasm are stained blue and red, respectively. **(B)** Confocal laser scanning microscopy confirmed the results obtained by fluorescence microscopy. Nuclei of the dead cells are stained orange whilst viable cells are stained green. **(C)** Quantitative analysis confirmed the results of microscopy analysis, data are represented as median with interquartile range, ^∗∗∗^*P* < 0.001, Mann–Whitney *U*-test.

Cell count after 6 days of culture revealed that HUVEC can be hardly cultured on both PHBV/PCL and PCL scaffolds without RGD peptides; however, modification of polymer surface with RGD peptides significantly enhanced endothelial cell viability (**Figures 5A,B**). The total number of cells on PHBV/PCL and PCL scaffolds with RGD peptides was 9.7- and 8.8-fold higher, respectively, compared to those without RGD peptides whilst the number of dead cells was similar (**Figure 5C**).

### RGD Peptides Recruit Endothelial Cells to the Implanted PHBV/PCL and PCL Vascular Grafts

**FIGURE 6:**
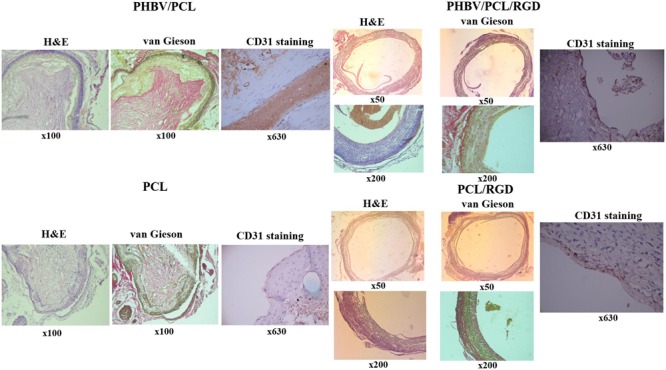
**Modification of PHBV/PCL and PCL vascular grafts with RGD peptides enhances formation of endothelial cell monolayer.** H&E and van Gieson staining revealed a putative endothelial cell monolayer on PHBV/PCL and PCL grafts with RGD peptides but not on those without; this was further confirmed by CD31 staining, which identified a monolayer of CD31-positive cells (brown) at the inner surface of both polymer grafts with RGD peptides.

Histological and immunohistochemical examination 1 month postimplantation revealed that both PHBV/PCL and PCL grafts without RGD peptides were occluded and did not contain any endothelial cells on their inner surface (**Figure 6**). However, we detected an endothelial cell monolayer in PHBV/PCL and PCL grafts with RGD peptides, which also were completely patent, thrombus-free, and had no signs of inflammation within the graft wall.

## Discussion

It has been demonstrated that RGD peptides enhanced cell adhesion as well as cell viability (de Mel et al., 2014). Covalent immobilization of RGD peptides on PCL surface led to an about 11-fold increase in endothelial cell attachment in comparison with untreated PCL (Gabriel et al., 2006). These results were further confirmed on another cell lines (Gabriel et al., 2012). RGD peptides improved haemocompatibility, cell infiltration, endothelialisation, formation of the smooth muscle cell layer, and patency of electrospun PCL vascular grafts implanted into rabbit carotid arteries for 2 and 4 weeks (Zheng et al., 2012). These results were further confirmed on PCL vascular grafts prepared by salt leaching (Kang et al., 2015). Moreover, it was shown that it is *in situ* recruitment of endothelial cells and endothelial progenitor cells but not *in vitro* endothelialisation that provides patency of the graft (Kang et al., 2015). Recently, Pontailler et al. (2015) demonstrated a successful formation of endothelial cell monolayer and continuous layer of smooth muscle cells on a poly(3-hydroxybutyrate-co-3-hydroxyvalerate-co-4-hydroxyvalerate) vascular graft modified with RGD peptides in a rat model of partial inferior vena cava replacement 6 weeks postimplantation.

Here we showed that modification of PHBV/PCL and PCL vascular grafts with RGD peptides alters their morphology, improves physico-mechanical properties, increases endothelial cell viability, and enhances graft endothelialisation *in vivo* 1 month postimplantation. PHBV/PCL grafts modified with RGD peptides had lower mean fiber diameter and pore area; however, this was opposite for PCL grafts, and it can be explained by a distinct nature of these polymers. RGD peptides decreased durability of both PHBV/PCL and PCL to almost similar to IMA; however, this was not the case for elasticity, compliance, and stress-strain curve. The results obtained in the cell culture experiments and in a rat model were similar for both polymers. To the best of our knowledge, there are no data regarding modification of PHBV/PCL vascular grafts with RGD peptides, particularly regarding model of arterial replacement. In addition, our results confirm those reported for PCL vascular grafts in the previous studies (Gabriel et al., 2012; Zheng et al., 2012; Kang et al., 2015) Therefore, modification of PHBV/PCL and PCL vascular grafts with RGD peptides improves their physico-mechanical properties and biocompatibility. The next stage of the research is using larger animal model for assessment of the bypass graft. We have already chosen the ovine model, which is the model of choice for the evaluation of cardiovascular implants *in vivo* (Rashid et al., 2004). The anatomic and haemodynamic conditions are sufficiently analogous to the human situation whilst the long neck region facilitates easy surgical access to vessels of appropriate size for implantation. Furthermore, sheep represent a “worst case model” due to the increased calcium metabolism allowing for the assessment of degenerative processes of cardiovascular implants in a relatively short time period (Hoerstrup et al., 2006).

## Author Contributions

LA, AS, AK, YK, OB, and LB conceived and designed the study; VV, EK, and ES fabricated the grafts; AS performed the experiments on RGD peptide modification; LA, VV, and TG performed morphological assessment; LA, AM, VV, and TG performed evaluation of physico-mechanical properties; LA, VV, EV, and VM performed *in vitro* experiments; AM, EV, EK, and EA performed *in vivo* implantation; LA, VV, EK, AB, and GV performed histological examination and immunohistochemistry; LA, AS, AK, and VV performed data analysis and wrote the manuscript.

## Conflict of Interest Statement

The authors declare that the research was conducted in the absence of any commercial or financial relationships that could be construed as a potential conflict of interest.

The reviewer AS and handling Editor declared their shared affiliation, and the handling Editor states that the process nevertheless met the standards of a fair and objective review.
